# Embedding Photoacids into Polymer Opal Structures: Synergistic Effects on Optical and Stimuli-Responsive Features

**DOI:** 10.3390/molecules26237350

**Published:** 2021-12-03

**Authors:** Martin Bitsch, Anna Katharina Boehm, Alexander Grandjean, Gregor Jung, Markus Gallei

**Affiliations:** 1 Polymer Chemistry, Saarland University, Campus Saarbrücken C4 2, 66123 Saarbrücken, Germany; martin.bitsch@uni-saarland.de (M.B.); annakatharina.boehm@uni-saarland.de (A.K.B.); 2 Biophysical Chemistry, Saarland University, Campus B2 2, 66123 Saarbrücken, Germany; alexander.grandjean@uni-saarland.de; 3 Saarene-Saarland Center for Energy Materials and Sustainability, Campus C4 2, 66123 Saarbrücken, Germany

**Keywords:** opal film, melt-shear organization, photoacid, excited-state proton transfer, fluorescence, polymer particle synthesis

## Abstract

Opal films with their vivid structural colors represent a field of tremendous interest and obtained materials offer the possibility for many applications, such as optical sensors or anti-counterfeiting materials. A convenient method for the generation of opal structures relies on the tailored design of core-interlayer-shell (CIS) particles. Within the present study, elastomeric opal films were combined with stimuli-responsive photoacids to further influence the optical properties of structurally colored materials. Starting from cross-linked polystyrene (PS) core particles featuring a hydroxy-rich and polar soft shell, opal films were prepared by application of the melt-shear organization technique. The photoacid tris(2,2,2-trifluoroethyl) 8-hydroxypyrene-1,3,6-trisulfonate (TFEHTS) could be conveniently incorporated during freeze-drying the particle dispersion and prior to the melt-shear organization. Furthermore, the polar opal matrix featuring hydroxylic moieties enabled excited-state proton transfer (ESPT), which is proved by spectroscopic evaluation. Finally, the influence of the photoacid on the optical properties of the 3-dimensional colloidal crystals were investigated within different experimental conditions. The angle dependence of the emission spectra unambiguously shows the selective suppression of the photoacid’s fluorescence in its deprotonated state.

## 1. Introduction

Starting with the early work of Eli Yablonovitch, photonic crystals became more and more popular because of their fascinating optical properties [1,2,3,4]. Photonic crystals are an analogue of semiconductors exhibiting a forbidden band gap for electromagnetic waves [5,6]. The basic prerequisites of such materials rely on a periodically differing refractive index within domain sizes that fit the wavelength of visible light [7,8]. Through interference with light, photonic crystals show their angle-dependent and vivid structural colors [9,10,11]. Since their discovery in the 1980s, many approaches for the fabrication of photonic crystals have been developed, and most of them are based on monodisperse nanoparticles [12,13,14,15,16]. As such, clusters of monodisperse silica particles can be found in natural opal gemstones, and photonic materials based on particles are called opal films [17]. The key feature to produce an opal film lies in the perfect arrangement of the underlying monodisperse particles. As those particles feature the capability of self-assembling into highly ordered structures, methods, such as vertical deposition or spin-coating, provide convenient ways for the opal film fabrication [18,19,20]. Another way to gain access to free-standing opal films is the so-called melt-shear organization [10,21]. For this method, core-interlayer-shell (CIS) particles with tailored properties are necessary: (i) the core material has to be inherently stable under increased temperature and applied shear forces, while (ii) the shell has to be soft and meltable. Due to the covalent connection between the shell and core (iii), the core particles assemble inside the shell material under application of moderate temperature (above the glass transition temperature of the shell material) and pressure. As a result of this process, free-standing opal films without any cracks can be fabricated after self-assembly of the monodisperse particles inside the viscoelastic matrix [22,23]. In the present work, we demonstrate the synthesis of CIS particles featuring hydroxylic moieties, which stem from different polar monomers, i.e., 2-(hydroxyethyl) methacrylate (HEMA) or isopropylideneglycerol methacrylate (IPGMA). After particle synthesis by means of emulsion polymerization protocols, the photoacid tris(2,2,2-trifluoroethyl) 8-hydroxypyrene-1,3,6-trisulfonate (TFEHTS) is added to the particle dispersion. Finally, freeze-drying and melt-shear organization lead to opal films loaded with the photoacid inside the opal matrix. A previous study focused on opal films comprising the fluorescent monomer rhodamine B methacrylamide, which showed an angle-dependent fluorescence suppression behavior [24]. Moreover, it is known that photoacids can be used for electrostatic self-assembly of cationic dendrimers according to their light induced negative charges, as well as in combination with block copolymers to change their hydrophobic/hydrophilic properties after UV-irradiation, which leads to a higher solubility in water [25,26].

**Scheme 1:**
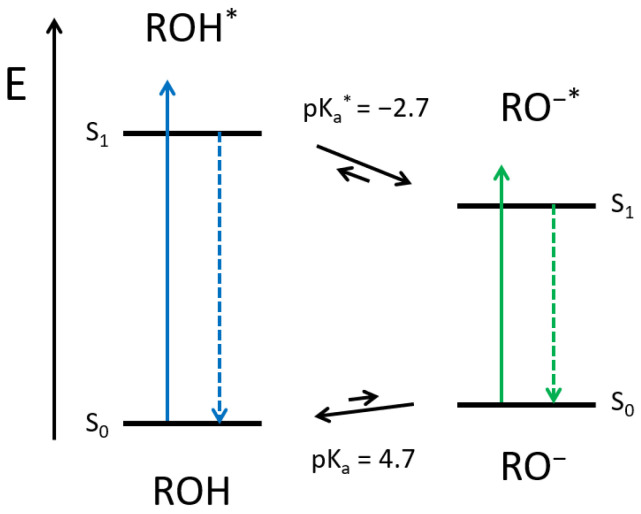
Energetic scheme of the here used photoacid tris(2,2,2-trifluoroethyl) 8-hydroxypyrene-1,3,6-trisulfonate (TFEHTS) and its pK_a_ values [34]. Solid lines represent absorption, and dashed lines denote fluorescence. The ROH* and RO^−^* are the protonated and deprotonated species of the photoacid in the exited state, respectively the pK_a_* signified the pK_a_ value in the exited state.

Here, photoacids are fluorophores, which become much more acidic upon electronic excitation. The peculiarity of photoacids compared to other strongly emissive molecules is that a photochemical reaction, i.e., the excited-state proton transfer (ESPT), competes with the radiative decay. Therefore, if the neutral form of the photoacid (ROH) is excited, and a suitable proton acceptor is available, the deprotonated species of the photoacid (RO^−^) is generated in the excited-state. Afterwards, the RO^−^ form fluoresces bathochromically, as the negative charge is stabilized through the aromatic system. Back in the ground state, the acidity drops again, and the photoacid becomes reprotonated (see Scheme 1). The reversible proton release (in the excited state) and recapture (in the ground state) is also known as the Förster Cycle [27]. However, solvatochromism and recent spectroscopic experiments highlighted the decisive impact of the surrounding in ESPT [28,29,30,31,32,33]. In the present case, two possible effects of the matrix as surrounding aroused our interest: (1) Can we exploit the reversible generation of charged molecules to manipulate the material photonic properties? (2) Can we manipulate the kinetics and, subsequently, the thermodynamics of a (photo)chemical reaction just by reversibly modifying the photonic structure?

The hydroxylic moieties, homogeneously distributed in the opal films’ matrix should be suitable proton acceptors and enable excited-state proton transfer and, thus, should pave the way for the design of a photoacidic polymeric photonic material (3P-material).

## 2. Results

**Figure 1:**
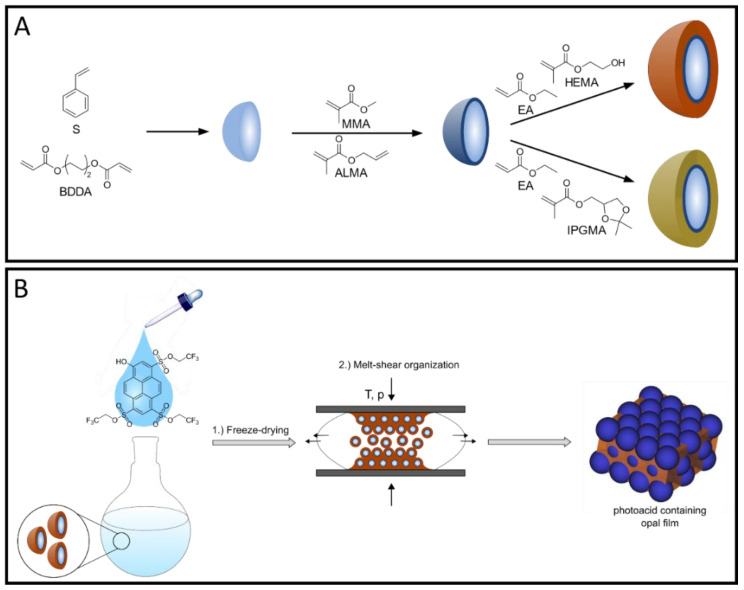
(**A**): Schematic representation of the synthesis of hard core poly(styrene-*co*-butanediol diacrylate) (P(S-*co*-BDDA)) and soft shell poly(ethyl acrylate-*co*-2-(hydroxyethyl) methacrylate) ((P(EA-*co*-HEMA)) or poly(ethyl acrylate-*co*-isopropylideneglycerol methacrylate) (P(EA-*co*-IPGMA))) particles by semi-continuous emulsion polymerization. The interlayer consists of poly(methyl methacrylate-*co*-allyl methacrylate) (P(MMA-*co*-ALMA)). (**B**): Incorporation of the photoacid (tris(2,2,2-trifluoroethyl) 8-hydroxypyrene-1,3,6-trisulfonate (TFEHTS)) and subsequent opal film preparation using the melt-shear organization technique.

For the fabrication of photoacid-containing opal films, monodisperse particles were synthesized using starved-feed emulsion polymerization (EP), homogenized with the respective photoacid TFEHTS, and subsequently transferred into the melt-shear organization process, as illustrated in Figure 1. For this purpose, it is necessary to synthesize particles with a CIS architecture with a cross-linked core that keeps its shape, even under increased temperature and shear forces during melt-shear organization. For the cross-linking reaction, the polystyrene (PS) core particles were copolymerized with the bifunctional monomer butanediol diacrylate (BDDA) comprising two reactive acrylate moieties. In addition, a soft shell with a low glass transition temperature (T_g_) is needed so that the polymer chains have sufficient mobility under increased pressure and temperature to form the opal film matrix, while the hard-core particles self-assemble into an ordered colloidal crystal structure.

The stepwise synthesis of the illustrated CIS particles was performed via seeded EP technique in a starved-feed mode. The first step comprised the synthesis of cross-linked poly(styrene-*co*-butanediol diacrylate) (P(S-*co*-BDDA)) core particles using a batch process, followed by the continuous addition of either S and BDDA, in a starved-feed mode. Thereby, the average diameter of the core particles was adjusted to be 203.2 ± 7.8 nm (according to dynamic light scattering (DLS) data). Using BDDA as the cross-linker reagent offers the possibility to reinitiate the polymerization via free vinylic moieties on the core particle surface. Thus, the cross-linked interlayer containing methyl methacrylate (MMA) and allyl methacrylate (ALMA) was introduced. Based on the two different reactive sites of ALMA, subsequent anchoring of the soft polymer shell material was possible. In this regard, it is well known from the literature that covalent grafting of the shell material, provided by an interlayer, is mandatory to prevent the detachment of the polymer chains and, simultaneously, ensure the particles’ stability [10,35,36].

**Figure 2:**
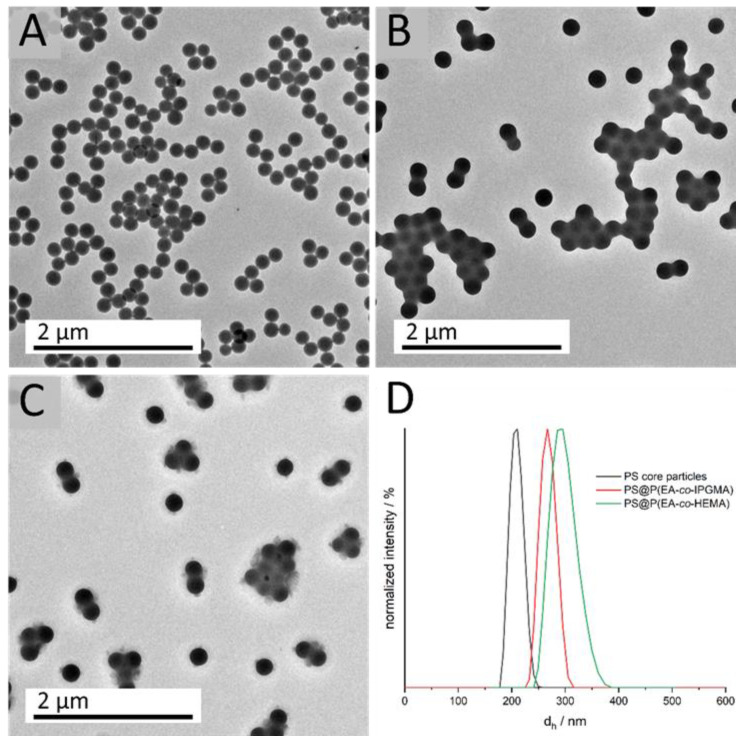
TEM images of (**A**) PS core particles, (**B**) PS@P(EA-*co*-IPGMA) particles, (**C**) PS@P(EA-*co*-HEMA) particles, and (**D**) DLS measurements of the core and the final CIS particles.

Concerning the melt-shear organization process, the so-called shear rate (η) is another important parameter, besides the aforementioned glass transition temperature. In this context, Finlayson et al. demonstrated that, above the T_g_, the poly(ethylacrylate) (PEA) shell shows viscoelastic properties with a corresponding shear rate of η = 10^3^–10^4^ Pa·s at 100 °C, which is important for the ordering process [37]. Moreover, protic polar environments turned out to enhance ESPT [29,30]; therefore, it is necessary to include hydroxylic moieties into the shell material. Furthermore, it can be assumed that, the higher the content of the hydroxylic groups in the copolymer, the higher the corresponding hydrophilicity. Based on these conditions, two strategies were used to obtain free-standing opal films, where a different content of hydroxylic moieties is located within the soft matrix material. On the one hand, 2 wt.% HEMA were introduced into the particle shell by EP, which led to the presence of free hydroxylic moieties inside the matrix material after melt-shear organization. The incorporation of HEMA via emulsion polymerization protocols is already known for additional thermally induced cross-linking reactions [35,38]. On the other hand, since HEMA contains only one hydroxylic group and more than 2 wt.% makes the final opal film brittle, isopropylideneglycerol methacrylate (IPGMA) with two hydroxylic groups protected by a ketal group was investigated. Based on the defined reaction conditions for the deprotection reaction, the protected polymer can be selectively transferred into two hydroxylic groups after opal film formation, which will be discussed in the subsequent section [39,40]. In addition, the protecting ketal group results in a lower water solubility compared to glycerol monomethacrylate (GMMA), which is beneficial during the emulsion polymerization. This is due to the fact that the more hydrophobic monomer does not enrich in the water phase and, consequently, leads to a more homogeneous growth on the core particles, resulting in an increased amount of IPGMA in the respective copolymer. To evaluate the successful preparation of tailored particles featuring a core-shell architecture, DLS and transmission electron microscope (TEM) measurements were carried out after each synthesis step. Corresponding DLS measurements, as well as TEM images, are given in Figure 2. 

DLS measurements of the diluted particle dispersions show a uniform size distribution, as well as an increasing hydrodynamic diameter d_h_, for the CIS particle synthesis, starting from 203.2 ± 7.8 nm for the cross-linked PS core particles to 268.3 ± 10.9 nm (PS@P(EA-*co*-IPGMA) and 296.0 ± 12.4 nm PS@P(EA-*co*-HEMA) for the final CIS particles. Furthermore, the small standard deviations of < 5% is a key feature for further processing and colloidal self-assembly, which is also proved by transmission electron micrographs. All obtained data on particle size were in good agreement with theoretical expectations to monomer consumption and polymer yield (see Experimental Section) and are summarized in Table 1.

For the calculation of the particle diameter in the dried state (TEM), 50 particles were measured. The small difference regarding the particle diameter, between DLS and TEM investigations, can be attributed to the fact that, in the case of DLS, the particles are characterized in a swollen state, resulting in a higher diameter d_h_ due to solvent interactions. Since the particles’ shell material is more hydrophilic compared to the cross-linked hydrophobic PS core particles, the hydrodynamic diameter is more distinct. The monodisperse distribution, which is a prerequisite for further processing, can also be concluded from the TEM images. Although the soft polymer shell tends to be influenced by the energy of the electron beam, the TEM images already indicate hexagonally ordering of the respective core shell particles. Considering the particles’ diameter with respect to the Bragg’s law of diffraction and an angle of view of 90°, a photonic bandgap or, rather, a Bragg-peak between 500–600 nm is expected.

**Figure 3:**
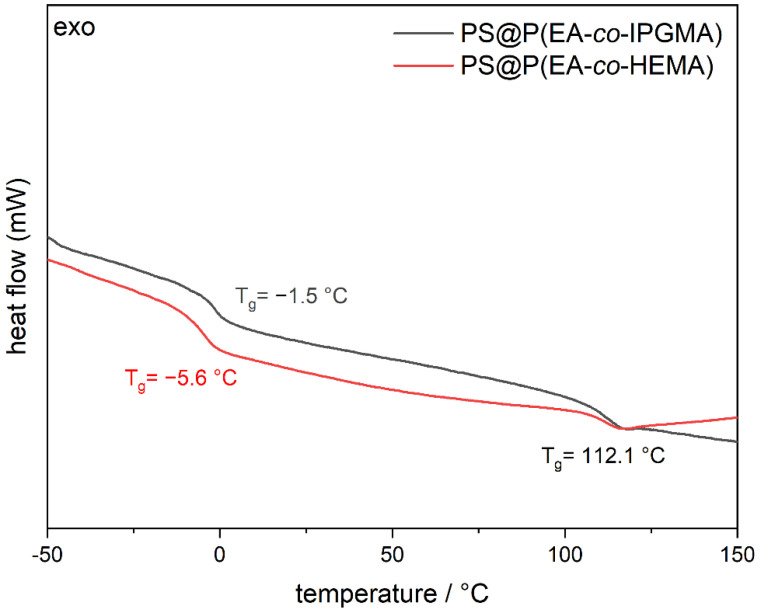
Differential scanning calorimetry (DSC) thermograms of the final PS@P(EA-*co*-HEMA), as well as PS@P(EA-*co*-IPGMA) core shell particles.

Another parameter of the materials that is of major importance, prior to the processing experiments, is the thermal properties of the CIS particles, especially for the shell material. The final T_g_ of the shell material determines the temperature of processing, on the one hand, and evaluates the successful incorporation of both monomers according to polymer consumption, on the other hand. Therefore, the T_g_ of the core shell particles was verified by differential scanning calorimetry (DSC) measurements, and the corresponding thermograms are illustrated in Figure 3.

**Figure 4:**
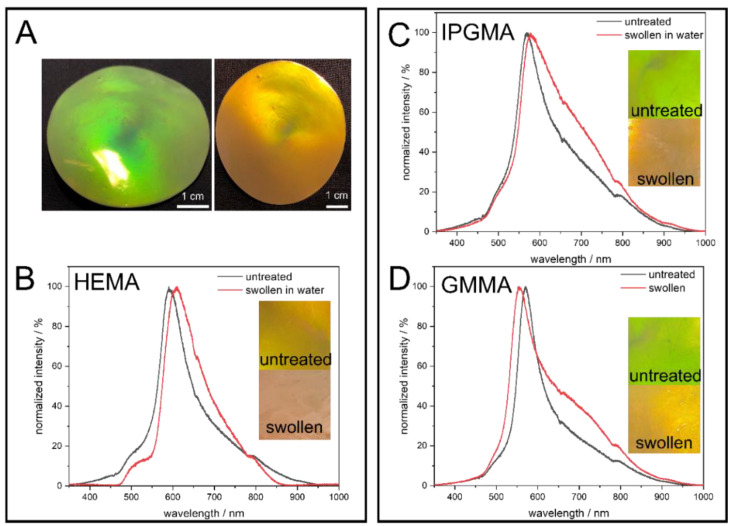
Photographs of (**A**) the PS@P(EA-*co*-HEMA) opal films under different angles of view, (**B**) as well as before and after swelling in water, and (**C**) the PS@P(EA-*co*-IPGMA) and (**D**) PS@P(EA-*co*-GMMA) opal films before and after swelling in water.

From Figure 3 it can be concluded that two glass transition temperatures were obtained for each CIS-particle batch, which is in good agreement with expectations from the synthesis protocols. In this regard, the cross-linked PS cores feature a glass transition at 112.1 °C since the cross-linking increase the corresponding T_g_ of polystyrene. Furthermore, the HEMA-containing particles show a glass transition at −5.6 °C conforming the successful copolymerization P(EA_98_-*co*-HEMA_2_) since the homopolymers have a Tg at −24 °C (PEA) [41] and 106 °C poly(2-(hydroxyethyl) methacrylate) (PHEMA) [42], respectively. In addition, the P(EA_85_-*co*-IPGMA_15_) copolymer shell revealed a T_g_ at −1.5 °C; hence, both CIS particle batches comply with the processing conditions for the preparation of photoacid-containing opal films via melt-shear organization. Besides the thermal properties, the respective monomers for the core and shell material were specifically chosen with respect to the effective refractive index to enable the generation of vivid structural colors according to Bragg’s law of diffraction. While PS has a refractive index of n_PS_ = 1.58, PEA has a relatively low refractive index of n_PEA_ = 1.47, resulting in a high refractive index contrast with vivid structural colors shown in previous works [35]. After the successful synthesis of well-defined CIS particles, both particle dispersions were homogeneously mixed with an ethanolic solution of the respective photoacid TFEHTS (final dispersion content 0.1 wt.-%) and subsequently freeze-dried. In the next step, the photoacid-containing particle powders were subjected to the melt-shear organization technique for the preparation of the respective opal films. As described in the introduction, by this method, free-standing photonic crystals can be obtained. Within the present study, during the shear-induced self-assembly, the cross-linked PS core particles were arranged into a random-closed packed structure surrounded by the soft polymer matrix containing the photoacid [37,43]. In order to optimize the brilliance of the colloidal crystal films, the particle dispersion was mixed with an aqueous carbon black solution (final solid content in the opal film 0.1 wt.-%) prior to freeze drying, to generate more scattering centers that subsequently enhance the structural color. Moreover, the opal films were incubated in deionized water to investigate the swelling behavior dependent on the polarity of the particle shell or rather the matrix material. The corresponding photographs under different angles of view, as well as before and after treatment with water, are given in Figure 4.

The images in Figure 4 clearly demonstrate the precise control over the particle synthesis since all colloidal crystal films show vivid structural colors due to interaction with visible light. To point out the angle dependency of the structural colors arising from the highly ordered nanostructure, photographs of the PS@P(EA-*co*-HEMA) opal film were exemplarily taken under different angles of view, showing a blue shift of the Bragg-Peak from red to green when the angle of view is decreased according to Bragg’s law of diffraction. At the same time, treating the opal film with water lead to a red shift, which can be seen here in a less pronounced reflection color caused. This behavior can be explained by the fact that the polar hydroxylic groups of PHEMA interact with the water molecules, followed by the swelling of the matrix material and, finally, lead to an increased lattice plane distance. Considering the PS@P(EA-*co*-IPGMA) opal film, the same behavior is observed, but the PS@(PEA-*co*-GMMA) opal film shows a shift toward smaller wavelength. This can be explained by the parameters of the Bragg equation (Equation (1)) and the refractive index contrast n_eff_. (Equation (2)).

λ_111_ = 2·a_111_·n_eff._·sin(δ), (1)

n_eff._ = ∑ϕ_i_ · n_i_. (2)

On one hand, swelling with water lead to an increase in the lattice plane distance a_111_ shifting the Bragg-peak to a higher wavelength, while, on the other hand, the refractive index of the matrix material changes during swelling, hence the refractive index contrast. Since water has a lower refractive index compared to all components present in the shell material, a higher swelling capability goes along with a higher water content and, thus, a lower n_eff_., shifting the Bragg-Peak to the opposite direction (lower wavelength). Conclusively, it can be said that the PHEMA, poly (isopropylideneglycerol methacrylate) (PIPGMA), and PGMMA-containing opal films prepared in this work show a water-responsive swelling behavior.

**Figure 5:**
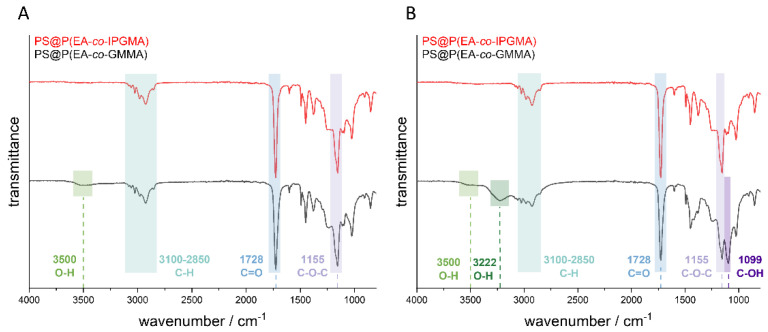
ATR-IR measurements of the synthesized opal films (**A**) with photoacid and (**B**) without photoacid.

Prior to the fluorescence spectroscopy measurements, the ketal protected hydroxy groups of the PIPGMA-containing opal film were transferred into the respective hydroxy moieties. For this purpose, the opal film was treated under hydrochloric conditions for at least three hours at 70 °C. The success of the deprotection was verified by ATR-IR spectroscopy investigations before and after treatment with hydrochloric acid (pH = 1). The corresponding ATR-IR spectra of the opal film with and without the photoacid are given in Figure 5.

Considering the spectra in Figure 5, the most significant peaks result from the ester group of the polymer. Thereby, the peak at 1728 cm^−1^ results from the C=O group and the peak at 1155 cm^−1^ from the C-O-C group. Furthermore, the peaks in the range between 3100–2850 cm^−1^ can be attributed to the C-H vibration of the CH_2_ and CH_3_ groups of the copolymer. In addition, it can be concluded that the sample containing the photoacid did not reveal a significant difference with respect to the shape of the spectrum between the protected PIPGMA and deprotected PGMMA, but a new peak at 3500 cm^−1^ became visible. This peak indicates an OH-group which should stem from the deprotection of the corresponding ketal group. Comparing the IR-spectra of the opal film without the photoacid, a more distinct difference was obtained after the deprotection, which can be observed at 3500 cm^−1^, 3222 cm^−1^, 1413 cm^−1^, and 1099 cm^−1^. While the signals at 3500 cm^−1^, 3222 cm^−1^, and 1413 cm^−1^ correspond to the O-H group, the signal at a wavenumber of 1099 cm^−1^ results from the C-OH group. The signal at 3500 cm^−1^ results from the free O-H vibration, which means that there are no hydrogen bonds between the hydroxy groups and the signal at 3222 cm^−1^ corresponding to the OH-groups which form hydrogen bonds to other hydroxy groups inside the opal film. This indicates a higher amount of deprotected sites within the opal film without the photoacid because the photoacid-containing opal film revealed only a signal at 3500 cm^−1^, which is caused by the free OH-group; therefore, a lower grade of deprotection is achieved. Furthermore, the signal at 1413 cm^−1^ represents the deformation vibration of the hydroxylic group. This, in turn, might be due the fact that the photoacid affects the deprotection since all remaining parameters were kept constant. Moreover, it has to be mentioned that PEA is the main component (85 wt.-%); hence, a lower deprotection grade only gives a significant O-H-signal at 3500 cm^−1^, while the remaining signals are overlapped by the excess of PEA vibrations.

**Figure 6:**
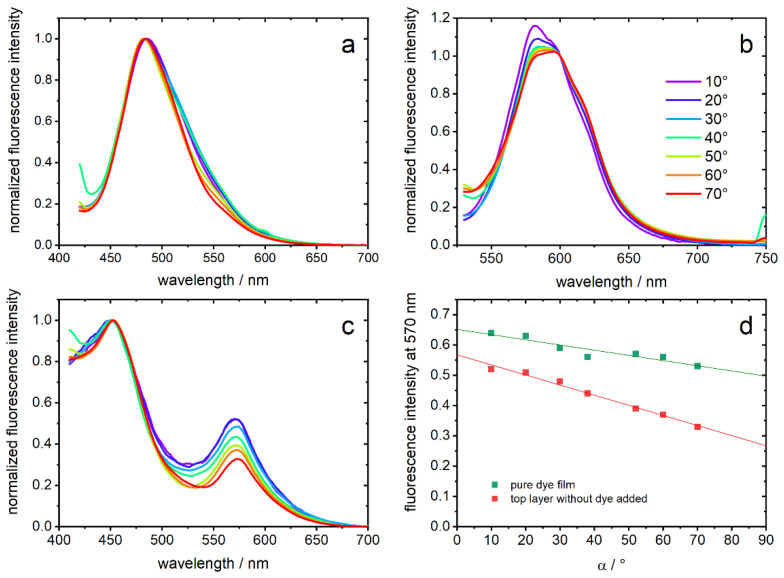
Emission spectra of polymer films with embedded dye: (**a**) PGMMA- (λ_exc_ = 400 nm), (**b**) HEMA- (λ_exc_ = 500 nm), and (**c**) PIPGMA-containing opal film (λ_exc_ = 380 nm) at indicated angles α between film surface and detection pathway. (**d**) The fluorescence intensity at 570 nm from (**c**) is plotted against α for a PIPGMA-containing opal film with and without a top layer of PIPGMA without dye.

For fluorescence measurements, the three opal films with embedded chromophore were covered with a top layer of the corresponding film without dye. Thereby, every emitted photon must pass the opal film structure. In Figure 6a–c, normalized emission spectra of the opal films are shown. They were measured depending on α, which is the angle between film surface and detection pathway. In PGMMA-containing opal-films, a fluorescence maximum of 482–485 nm (depending on the angle, λ_exc_ = 380 nm) is detected and can be assigned to the neutral photoacid (ROH), which is the dominant species in this matrix. The observed emission indicates a rather polar environment on the basis of the known solvatochromism [28]. In contrast, opal films comprising HEMA excited with 500 nm show an emission maximum at 581–595 nm, which is expected for the deprotonated form (RO^−^) [28,34]. Excitation with 400 nm shows only a small contribution of ROH (data not shown). Regarding the PIPGMA-containing opal-film, two maxima of 452 nm and 572 nm are observed and can be assigned analog to ROH and RO^−^. These hypsochromic shifts can be explained by the more apolar environment compared to the other films [28]. It should be noted that no fluorescence was detected without addition of photoacids.

Looking at the angle dependencies, a higher angle α results in a decrease of the fluorescence signal between 500–700 nm in all three opal films. We interpret this observation, common to all three samples which only share the core particle from the same batch, as the interaction of the opal structure with the emitted light [44,45]. While the impact of the photonic structure in Figure 6a,b leads to a deformation of the emission spectra in the region of the expected optical band gap, PIPGMA appears especially appropriate to study the interplay of the material with the emission of the photoacids: an intensity ratio is inherently insensitive to varying excitation intensity and, thus, more reliable to judge optical effects. To demonstrate, hence, the effect of the photonic material, the fluorescence intensity of PIPGMA-containing opal films at 570 nm (Figure 6c) is plotted against α in Figure 6d. For PIPGMA colloidal crystals without a non-fluorescent top layer, the angle dependency seems to be less sensitive and less suppressed than for a PIPGMA-containing opal film, covered with an additional non-fluorescent top layer. The linear fits in Figure 6d do not rely on any underlying physical models but quantitatively show the dependence of the fluorescence from the detection angle. The slope changes from −17(±3) × 10^−4^ to −33(±2) × 10^−4^ by adding the additional photonic layer without dye. This means that the fluorescence band at 570 nm is angle-dependent suppressed, e.g., by the particle-organization. Probably, fluorescence from dye molecules on the surface, whose emitting photons have no, or only a short, pathway through the structured film, mask the angle dependence. This is excluded by adding the corresponding non-fluorescent top layer.

**Figure 7:**
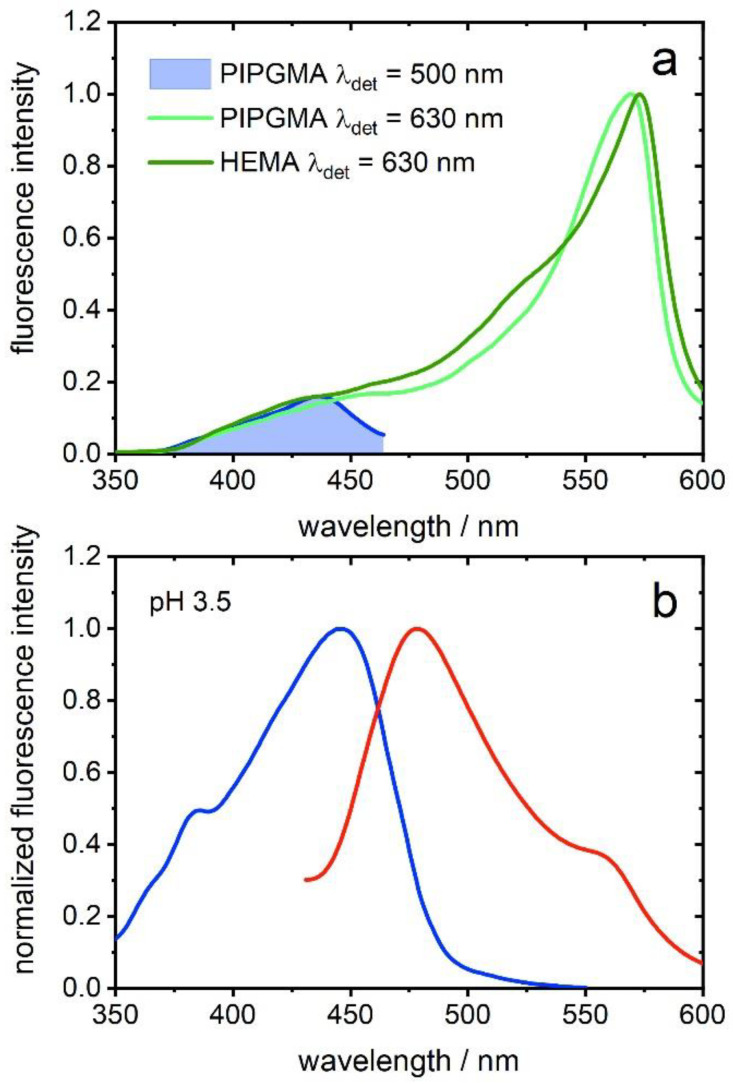
(**a**) Excitation spectra of PIPGMA- and HEMA-containing opal films at indicated detection wavelength λ_det._; (**b**) excitation (λ_det_ = 600 nm) and emission spectrum (λ_exc_ = 400 nm) of a PGMMA-containing opal film swollen in water (pH 3.5) for 72 h.

The remaining question is if the RO^−^ emission band originates from excitation of ROH, e.g., ESPT, or from absorption of RO^−^. In the case of PHEMA-containing opal films, RO^−^ seems to already be the dominant species in the ground state. The excitation spectra show only a small shoulder at around 450 nm, which likely is traced back to excitation of the ROH species, and a maximum at 565 nm (Figure 7a). PIPGMA-containing opal film shows a similar excitation spectrum (Figure 7a), hinting to observed ESPT. For confirmation, a PGMMA-containing opal film was swelled in water (pH 3.5) for 72 h to inject proton acceptors for ESPT in a system with mainly ROH species. As shown in Figure 7b, the emission spectrum of a watered PGMMA-containing opal film shows an additional shoulder at around 560 nm, which can be assigned to RO^−^ [34]. On the other hand, the excitation spectrum exclusively shows a band at 447 nm, which is assigned to ROH. Although the extent of emission of the conjugated base of the photoacid is minor, its observation unambiguously points to ESPT [30]. Summarizing, PHEMA- and PIPGMA-containing opal films spectra show evidence for ESPT, which was confirmed for a PGMMA-containing film by adding water (pH 3.5) as proton acceptor.

## 3. Materials and Methods 

Ethyl acrylate (EA, 99.5%), 2-(hydroxyethyl) methacrylate (HEMA, 97%), methyl methacrylate (MMA, 99%), allyl methacrylate (ALMA, 98%), butanediol diacrylate (BDDA, 90%), styrene (S, ≥99%), DL-1,2-isopropylideneglycerol (≥97%), and triethylamine and methacryloyl chloride (≥97%) were purchased from Sigma Aldrich (St. Louis, MO, USA). Before the emulsion polymerization, the radical inhibitors were removed from the monomers by passing through an alumina column (basic, 50–200 µm). Potassium hydroxide flakes (90% reagent grade), sodium disulfite (for analysis), sodium persulfate (≥98%), Igepal CO-520, and sodium dodecyl sulfate (≥ 98.5%) were purchased from Sigma Aldrich, and Dowfax 2A1, a surfactant having a dual polar sulfonate head group and a non-polar C12 alkyl chain, from Dow Chemicals (Midland, MI, USA). For use in emulsion polymerization, deionized water was degassed by a constant nitrogen flow for at least 30 min. Triethylamine was dried over calcium hydride (CaH_2_) and freshly distilled before further used. All other chemicals were purchased from Sigma Aldrich and VWR (Radnor, PA, USA) and used as received, if not otherwise mentioned.

Dynamic light scattering (DLS) measurements of the particle dispersions (solid content of 0.12 ppm, 1.2 × 10^–7^ wt.-%) were performed with a Zetasizer ZS90 (Malvern Instruments, Malvern, UK) equipped with a 4 mW, 633 nm HeNe Laser. Measurements were carried out at 25 °C at an angle of 90° with a five-fold determination of 15 runs and an equilibration time of 120 s. Automated data acquisition was carried out using a cumulant fit. For the calculations, the z weight average hydrodynamic diameters were used.

Transmission electron microscopy (TEM) was done with a JEOL JEM 2100 electron microscope (Akishima, Präfektur Tokio, Japan) at an operating voltage of 200 kV. To investigate polymer-based particles, diluted dispersions were drop casted on a nitrocellulose coated copper grid. The shown TEM images were recorded in bright field mode by a Gatan Orius SC1000 CCD camera (Gatan, Pleasanton, CA, USA). The control of the camera was computer resisted using Gatan Microscopy Suite software (AMETEK, Inc., Berwyn, PA, USA).

For evaluation of the thermal properties of the synthesized CIS particles, differential scanning calorimetry (DSC) was carried out with a DSC 214 F1 Polyma from Netzsch (Selb, Bavaria, Germany) in a temperature range of −80 to +150 °C and a heating rate of 10 K min^−1^ in a nitrogen atmosphere.

Reflection spectra were recorded using a fiber FLAME T VIS NIR ES spectrometer from Ocean Optics (Ostfildern, Germany) combined with a high-powered tungsten halogen lamp HL 2000 HP FHSA (output power 20 W) and a 400 µm premium fiber QP400 2 VIS BX.

Fluorescent spectra were measured with a JASCO FP 6500 spectrofluorometer (JASCO incorporated, Easton, MD, USA) and a custom-made sample holder for the films. The sample holder allowed to adjust the angle α between film surface and detection pathway between 10° and 70°. If not mentioned otherwise, a top layer without dye was added to the actual film. Microscope cover slips of 170-μm thickness were used as carrier material of the films. Spectra were normalized to the fluorescence maximum of one of the emissive species or shown angles to cope with the varying excitation intensities and for the sake of a better comparability.

UV-vis measurements were carried out using a fiber FLAME T VIS NIR ES spectrometer from Ocean Optics, including a high-powered tungsten halogen lamp HL 2000 HP FHSA (output-power: 20 mW) and a 400 µm premium fiber QP400 2 VIS BX. All spectra were recorded in reflectance.

### 3.1. Synthesis of Isopropylideneglycerol Methacrylate (IPGMA)

100 mL of dry toluene, 12.1 mL triethylamine, and 10.03 mL DL-1,2-isopropylideneglycerol were transferred into a heated 500 mL round bottom flask under inert gas atmosphere and subsequently cooled to 0 °C. In the next step, 5.95 mL methacryloyl chloride were slowly added within 1 h and stirred over night at room temperature. After filtration, the light-yellow solution was extracted three times with deionized water to remove any unreacted educt and the organic phase dried with sodium carbonate. Finally, the solvent was evaporated under reduced pressure, giving isoproylideneglycerol methacrylate as a colorless oil.

### 3.2. Synthesis of Cross-Linked Polystyrene Core Particles

Cross-linked polystyrene (PS) core particles were synthesized in a 5 L double-wall reactor equipped with a stirrer and a reflux condenser under an argon atmosphere at 75 °C. For this purpose, the vessel was filled with a monomer emulsion (ME) of 18 g S, 2 g butanediol diacrylate (BDDA), 1400 g deionized water, and 1.2 g sodium dodecyl sulfate (SDS) and stirred at 300 rpm. The polymerization was subsequently initiated by the addition of 0.18 g sodium disulfite (NaDS), 2.59 g sodium persulfate (NaPS), and 0.18 g NaDS (each component was dissolved in 5 g of deionized water). After a 15-min reaction time, a monomer emulsion (ME1) containing 1.67 g SDS, 2.92 g potassium hydroxide (KOH), 1.6 g Dowfax 2A1, 513 g S, 51.3 g BDDA, and 659 g deionized water was added continuously with a flow rate of 6 mL min^−1^ using a rotary piston pump reglo-CPF digital, RH00 (Ismatec, Wertheim, Germany). After complete addition of ME1, the reaction was kept at a constant temperature for an additional 60 min, resulting in a solid content of the particle dispersion of 16.4 wt.-%, respectively. The average hydrodynamic diameter of the cross-linked PS core particles was 203.2 ± 7.8 nm, determined by dynamic light scattering (DLS).

### 3.3. Synthesis of HEMA-Containing CIS Particles

The HEMA-containing CIS particles were synthesized starting from the PS core particle dispersion (particle size 203.2 ± 7.8 nm) in a 250 mL double-wall reactor equipped with a stirrer and a reflux condenser under a nitrogen atmosphere at 75 °C. For this purpose, 27.39 g of the PS core dispersion and 28.17 g deionized water were filled into the reactor and stirred at 280 rpm. The polymerization was initiated by the addition of 0.006 g NaDS, 0.069 g NaPS, and 0.006 g NaDS (each component was dissolved in 5 g of deionized water). After a 15-min reaction time, a monomer emulsion (ME1) consisting of 0.007 g SDS, 0.014 g Dowfax 2A1, 0.61 g methyl methacrylate (MMA), 0.068 g allyl methacrylate (ALMA), and 3 g deionized water was continuously added with a flow rate of 0.2 mL min^−1^ using a rotary piston pump. After the complete addition of ME1 and an additional 20 min stirring at this temperature, the polymerization was reinitiated by adding 0.011 g NaPS. After an additional 10 min, a second monomer emulsion (ME2), containing 0.087 g Igepal CO-520, 0.046 g Dowfax 2A1, 0.08 g KOH, 14.1316 g ethyl acrylate (EA), 0.29 g HEMA, and 18.8 g deionized water, was added continuously with a flow rate of 0.2 mL min^−1^ using a rotary piston pump. After the complete addition of ME2, the reaction was kept at a constant temperature for an additional 60 min, resulting in a solid content of the CIS particle dispersion of 17.57 wt.-%. The average hydrodynamic diameter of the final CIS particles was 296.0 ± 12.4 nm determined by dynamic light scatting.

### 3.4. Synthesis of IPGMA-Containing CIS Particles

For the synthesis of IPGMA-containing core shell particles, 31.25 g of the PS core particle dispersion and 33.75 g deionized water were placed in a 100 mL double-wall reactor equipped with a stirrer and a reflux condenser under an argon atmosphere and heated up to 85 °C. Then, 7 mg of NaDS and 50 mg of NaPS were added. After a 15-min reaction time, a monomer Emulsion (ME1) consisting of 50 mg KOH, 430 mg EA, 75 mg ALMA, 13 mg Dowfax 2A1, and 6 g of deionized water was continuously added with a flow rate of 0.14 mL min^−1^ using a rotary piston pump. After the complete addition of the ME1 and an additional 20 min of stirring at 85 °C, a monomer emulsion (ME2) consisting of 10 mg Triton, 5 mg SDS, 825 mg IPGMA, 4675 mg EA, and 8 g deionized water was continuously added with a flow rate of 0.14 mL min^−1^ using a rotary piston pump. After 2 h, 50 mg of NaPS was added to the dispersion. After the complete addition of ME2, the reaction was kept at a constant temperature of 85 °C for an additional 60 min. The average hydrodynamic diameter of the final CIS particles was 268.3 ± 10.9 nm, determined by dynamic light scatting.

### 3.5. Deprotection of PS@P(EA-co-IPGMA)

The whole opal film was placed in 200 mL beaker. Then, 100 mL of a hydrochloric acid solution (pH = 1) was added and heated up to 70 °C for at least 3 h. After cooling to room temperature, the opal film was washed several times with deionized water and dried at room temperature overnight.

## 4. Conclusions

Within the present manuscript, we investigated an efficient strategy to generate highly ordered and free-standing colloidal crystal films incorporated with a photoacid, which allows for studying photoacidity in photonic materials, for the first time. For the opal film preparation, the melt-shear organization technique was applied using a specific CIS-particle architecture based on cross-linked PS cores and a hydroxyl-containing polymer shell. In this regard, PEA was used for brilliant optical properties, while PHEMA or PGMMA imply the corresponding proton acceptor functionalities. The precise control over the starved-feed EP was verified by TEM and DLS, as well as DSC, measurements according to particle size, size distribution, polymer consumption, and thermal properties. In addition, the photoacid was sufficiently incorporated by freeze-drying the respective mixture and transferred into opal films with angle dependent structural colors via melt-shearing. Subsequently, the obtained opal films were investigated by their optical properties according to Braggs law of diffraction. As a proof of principle, the herein synthesized materials were additionally characterized for the integration of the photoacid and the mutual influence on each other. On the one hand, the provoked ESPT is an elementary chemical reaction which is ideally suited to investigate the influence of quantum optical phenomena on chemistry. On the other hand, the photochemical generation of charged species is foreseen to lead to a swelling of the polymeric material which should alter the optical properties of this soft material. Clearer signatures of the interaction of the optical band structure were observed when the fluorescent layer was covered by an additional photonic layer: Fluorescence spectra revealed an angle dependency in the expected region of 500–700 nm. Furthermore, ESPT was demonstrated in the photonic material. For application and unequivocal manipulation, further optimizations, such as the covalent incorporation and subsequent exposure to polar solvents, are required.

## Figures and Tables

**Table 1:** Summary of the average particle diameter after each synthesis step determined by DLS and TEM.

Sample	d_h, DLS_/nm	d_TEM_/nm
PS core particles	203.2 ± 7.8	198.6 ± 9.6
PS@P(EA-*co*-HEMA)	296.0 ± 12.4	222.9 ± 8.3
PS@P(PEA-*co*-IPGMA)	268.3 ± 10.9	223.7 ± 11.4

## Data Availability

The data presented in this study are available on request from the corresponding authors.

## Sample Availability

Samples of the compounds are available from the authors.
